# Bisphenol A and DDT disrupt adipocyte function in the mammary gland: implications for breast cancer risk and progression

**DOI:** 10.3389/fonc.2025.1490898

**Published:** 2025-02-17

**Authors:** Sarah M. Bernhardt, Carrie D. House

**Affiliations:** ^1^ Department of Biology, San Diego State University, San Diego, CA, United States; ^2^ Moores Cancer Center, University of California, San Diego, La Jolla, CA, United States

**Keywords:** bisphenol A, dichlorodiphenyltrichloroethane, environmental obesogens, mammary adipocytes, breast cancer

## Abstract

As breast cancer incidence continues to rise worldwide, there is a pressing need to understand the environmental factors that contribute to its development. Obesogens, including Bisphenol A (BPA) and Dichlorodiphenyltrichloroethane (DDT), are highly prevalent in the environment, and have been associated with obesity and metabolic dysregulation. BPA and DDT, known to disrupt hormone signaling in breast epithelial cells, also promote adipogenesis, lipogenesis, and adipokine secretion in adipose tissue, directly contributing to the pathogenesis of obesity. While the adipose-rich mammary gland may be particularly vulnerable to environmental obesogens, there is a scarcity of research investigating obesogen-mediated changes in adipocytes that drive oncogenic transformation of breast epithelial cells. Here, we review the preclinical and clinical evidence linking BPA and DDT to impaired mammary gland development and breast cancer risk. We discuss how the obesogen-driven mechanisms that contribute to obesity, including changes in adipogenesis, lipogenesis, and adipokine secretion, could provide a pro-inflammatory, nutrient-rich environment that promotes activation of oncogenic pathways in breast epithelial cells. Understanding the role of obesogens in breast cancer risk and progression is essential for informing public health guidelines aimed at minimizing obesogen exposure, to ultimately reduce breast cancer incidence and improve outcomes for women.

## 1 Introduction

Breast cancer is a heterogenous disease, which can be classified into different subtypes based on a tumor’s histological and molecular characteristics, including hormone receptor (HR) status and gene expression profile (1). Triple-negative breast cancers (TNBC) are an aggressive subtype, characterized by a lack of estrogen receptor (ER), progesterone receptor (PR) and human epidermal growth factor receptor 2 (HER2) expression. TNBC comprise ~10-20% of all breast cancers (2), and are associated with worse prognosis relative to HR-positive breast cancer (3, 4).

As breast cancer incidence continues to rise worldwide, there is a pressing need to understand the environmental factors that contribute to its development. Recent evidence has implicated obesity as a risk factor for breast cancer (5, 6); however, the relationship between obesity and breast cancer subtype is complex. Among premenopausal women, obesity is strongly associated with an increased risk of TNBC, and suggested to decrease the risk of HR-positive disease (7–9). Further, obesity is more common in young women with TNBC, compared to women with HR-positive disease (10–12). In contrast, in postmenopausal women, obesity is more strongly linked to HR-positive tumors (9). While obesity has been associated with HR-positive disease in older women, the relationship between obesity and TNBC in young women is particularly concerning due to its poor prognosis and lack of targeted therapies. The biological mechanisms associated with obesity might also drive the development of more aggressive, triple-negative breast cancer in young women, suggesting obesity as a modifiable risk factor for young women’s breast cancer.

While the causes of obesity are complex, recent studies implicate ‘obesogens’ as playing a key role in their pathogenesis. Obesogens are environmental chemicals that disrupt hormonal regulation and metabolic processes, and are associated with an increased susceptibility for obesity and related metabolic disorders (13–18). Specifically, obesogens increase fat accumulation by promoting adipogenesis, disrupting fatty acid metabolism, and altering adipokine secretion, which can affect satiety signals and disrupt insulin and glucose regulation. However, given the propensity of obesogens to accumulate in fat, and their effects on adipocyte function, the adipose-rich mammary gland might be particularly prone to the effects of obesogens. Indeed, there is growing evidence that exposure to obesogens affects mammary gland development, and may increase the risk of developing breast cancer (19–23).

Despite the proposed association between obesogen exposure and breast cancer risk, the relationship between obesogens and the development of specific breast cancer subtypes are controversial. Global trends indicate that the rise in breast cancer incidence is largely driven by an increase in HR-positive disease (24). The concurrent rise in obesity suggests that obesogens may promote development of HR-positive disease. However, this relationship is controversial, as while obesity is strongly linked to HR-positive disease in postmenopausal women, it increases the risk of TNBC in premenopausal women. Differences in breast cancer subtypes may be driven by variations in hormonal and metabolic environments. In support of this, in breast cancer patients, obesity alters tumor metabolism in a subtype-specific manner, impacting both tumor behavior and outcomes (25).

While the relationship between obesogens and specific breast cancer subtypes remains unclear, there is evidence to suggest that obesogens might promote the development of more aggressive disease. Obesogens influence adipogenesis and lipid metabolism, and alter the adipokine milieu, resulting in a pro-inflammatory and nutrient-rich environment (26). These environmental conditions may be particularly supportive of the initiation and progression of TNBC, which is characterized by increased proliferation and chronic inflammation (27). Further, obesity itself is a risk factor for TNBC in young women, indirectly linking obesogen exposure to an elevated risk of TNBC. Based on these observations, we suggest that the impact of obesogens on the adipose-rich mammary gland may favor the development of TNBC, compared to less aggressive subtypes.

In this review, we examine the impact of common environmental contaminants, Bisphenol A (BPA) and Dichlorodiphenyltrichloroethane (DDT), on breast cancer risk, with a particular focus on how these obesogens affect mammary adipocytes and their paracrine interactions with the mammary epithelium. While this review focuses on the effects of BPA and DDT due to their extensive environmental presence, other prevalent obesogens pose health risks. Chronic exposure to obesogens, including phthalates (found in plastics and cosmetics), organotins (used as pesticides and in disinfectants), and perfluorooctanoic acids (used in non-stick coatings and water-resistant materials) (28) may similarly influence breast cancer risk through related mechanisms.

BPA is a synthetic chemical widely used in the production of polycarbonates and epoxy resins (29). BPA is found in numerous everyday items, including plastic bottles, food containers, cosmetics, and medical devices (30). In 2022, global production of BPA reached 8 million tons (31, 32), emphasizing its extensive presence in consumer products. This widespread use has led to routine human exposure through ingestion, inhalation, and direct skin contact (33, 34), as well as significant environmental contamination. Continued exposure to BPA presents considerable health risks that warrant further attention (35). Critically, BPA can cross the placenta (36), and has been detected in breast milk (37), which raise concerns surrounding its impact on fetal and infant development.

DDT is another prevalent environmental contaminant, with significant implications for human health. Discovered in 1939, DDT was widely used as an insecticide in the US until 1972, when concerns over environmental and health impacts led to widespread bans. To date, the World Health Organization continues to support use of DDT in some African and Asian countries to combat malaria, due to its role as an insecticide (38). Despite being banned, these chemicals continue to pose environmental and health risks today. Their long half-lives lead to bioaccumulation in the adipose tissue of animals (39), and persistence in environmental reservoirs such as soil and water (40). Further, DDT can cross the placenta to enter fetal circulation (41), and is found in high concentrations in breast milk in exposed mothers (42, 43), raising concerns for the health of offspring.

In this review, we discuss how BPA and DDT influence adipogenesis, lipogenesis, and adipokine secretion in mammary adipose tissue, and how this could potentially contribute to the development of breast cancer, with a focus on TNBC. The lack of estrogen receptor expression on TNBC suggests that obesogen-driven effects may be mediated by paracrine signaling within the surrounding microenvironment. Further, treatment of TNBC poses significant challenges due to its aggressive phenotype and lack of therapeutic targets. Understanding the impact of obesogens on TNBC development is essential for guiding policies to reduce exposure, with the potential to not only prevent TNBC progression, but also address the broader effects of these environmental contaminants on cancer and metabolic disorders.

## 2 Environmental obesogens BPA and DDT are associated with obesity

Obesogens are suggested to play a key role in the pathogenesis of obesity. Obesogens disrupt normal metabolic functions, contributing to the formation of new adipocytes, increased lipid accumulation, and altered energy use, thereby increasing the risk of obesity and related metabolic disorders (44). Among these, BPA and DDT are associated with impaired metabolic function and an increased risk of obesity in humans (13–18) and in rodent models (45–48), with early-life exposure resulting in more pronounced effects (22, 23).

Early life exposure to BPA is suggested to influence long-term health outcomes, particularly obesity and metabolic disorders in adults. In rodents, exposure to BPA in the perinatal period—a period which encompasses both *in utero* development and the postnatal phase—is associated with increased body weight and obesity in adult offspring (45, 46, 48). In children and adolescents, exposure to BPA, measured by concentrations in urine or serum, associates with an increased risk of metabolic syndrome, type 2 diabetes, and obesity, as defined by increased body mass index (BMI), fat mass, and/or waist circumference (13, 14, 18). Together, these observations raise concerns that early-life exposure to BPA may predispose individuals to metabolic disorders and obesity later in life. However, in adults, concentrations of BPA also associate with an increased risk of obesity and metabolic disorders (13, 14, 18), suggesting that chronic exposure during adulthood may similarly contribute to these health outcomes.

Similar to BPA, exposure to DDT—and its primary metabolite dichlorodiphenyldichloroethylene (DDE)—are associated with obesity. In rodent models, perinatal exposure to DDT elevates serum DDT and DDE to concentrations comparable to levels observed in humans, and associates with increased body weight of offspring (47). In humans, maternal DDT and DDE concentrations in serum (15, 17) and adipose tissue (16) associate with increased BMI in offspring. Together, these results demonstrate that early-life exposure to obesogen DDT can have profound long-term effects on obesity. While much of the research surrounding DDT/DDE on obesity investigates early-life exposure, it is possible that cumulative exposure to these obesogens during adulthood similarly contributes to long-term metabolic health risks.

## 3 Obesogen exposure impairs mammary gland development and increases breast cancer risk

The impact of obesogens on human health extends beyond obesity, with emerging concerns on their potential effects on the mammary gland. While obesogens primarily act as xenoestrogens to disrupt estrogen signaling in mammary epithelium, they also influence mammary gland signaling through other hormone-related pathways, including thyroid receptors and glucocorticoid receptors (49, 50). Further, obesogens interact with steroidogenic enzymes, resulting in impaired steroid hormone synthesis and regulation (49, 50), and can induce epigenetic changes, including DNA methylation (49, 50). Together, the diverse effects of obesogens could collectively disrupt mammary gland development and contribute to an increased breast cancer risk.

BPA and DDT have been shown to disrupt metabolic processes in adipose tissue, including increasing inflammatory cytokine production and altering lipid metabolism (51). Adipocytes comprise a large proportion of the mammary gland, primarily white adipocytes that function in lipid storage and hormone signaling. During pregnancy and lactation, white adipocytes reversibly transdifferentiate into pink adipocytes (52), which function to support milk production. Critically, these adipocytes in the mammary gland are sensitive to estrogens (53, 54) and have been shown to play a critical role in mammary gland development (55). While much of the current research has focused on the effects of obesogens on mammary epithelium, the adipose-rich mammary gland may be particularly vulnerable to obesogens. In support of this, studies suggest that BPA and DDT affect mammary gland development, and potentially increase breast cancer predisposition later in life.

In rodents, perinatal exposure to BPA perturbs mammary gland development (56), through increasing terminal end bud number and lateral branching during puberty (57). Further, *in utero* exposure to BPA increases total alveolar bud area and total epithelial structures in adult offspring (58). These changes in mammary development may leave the gland susceptible to neoplastic development. Indeed, preclinical studies demonstrate that perinatal BPA exposure results in a heightened responsiveness to estrogen during puberty (57, 59). Further, BPA exposure during prenatal development accelerates hyperplasia in rodent models (60–62). Together these data suggest that early exposure to BPA enhances the mammary gland’s sensitivity to estrogen, leading to an increased risk and earlier onset of mammary cancer. While these effects are primarily epithelial, epithelial-adipocyte interactions are crucial for mammary gland development. It remains unclear whether BPA-induced changes in adipocyte function contribute to the observed changes in mammary gland development, and whether such changes can influence cancer risk or promote the development of specific breast cancer subtypes.

Epidemiological studies suggest that exposure to BPA, measured by increased concentrations in urine (19–21) or breast adipose tissue (20), is associated with an increased risk of breast cancer development. However, other studies found a lack of association (63–68), leaving this relationship controversial. These conflicting results may stem from several factors. Measurement of BPA in urine or adipose tissue often occurred after cancer diagnosis, with some studies collecting samples prior to adjuvant therapy (19, 21, 66), while other studies included pre- and post-treated samples (65), or did not report chemotherapy exposure (20, 67, 68). Further, while some studies considered factors including menopausal status, reproductive history, and obesity (65, 66, 68), other studies did not account for these variables (19, 20, 67). Another limitation in current research is that BPA measurements are based on single time-point samples collected from older women, and are unable to assess the potential effects of BPA exposure during critical periods of breast development. Preclinical models suggest that the effects of BPA on tumor progression are amplified in early development (57, 59), emphasizing the importance of understanding how early-life exposure contributes to breast cancer risk.

Exposure to DDT/DDE has also been linked to altered mammary gland development and increased breast cancer risk. In rodents, DDT exposure in drinking water increases terminal end bud proliferation and accelerates lobule differentiation (69); effects that could predispose the mammary gland to carcinogenic transformation. In mice, DDT treatment promotes growth of estrogen-responsive tumors (70). In *MMTV*-*Neu* mice, implantation of DDE pellets in the mammary fat-pad, which mimic the propensity of DDE to accumulate in adipose tissue, significantly accelerates tumor development (71). Together, these studies suggest that DDT/DDE similarly acts on epithelial cells to increase mammary cancer risk. However, it remains unclear whether DDT/DDE also impacts adipocyte function during mammary gland development, and whether such effects contribute to mammary cancer susceptibility or the development of specific subtypes.

While some meta-analyses report that increased serum DDT and/or DDE concentrations associate with increased breast cancer risk (22, 23, 64); other analyses found no association (72–74). This controversy could be due to the heterogeneity of studies included in the meta-analyses, where variability in sample collection methods (serum, tissue, urine), differences in exposure to adjuvant therapy (pre- versus post-chemotherapy), and variability in accounting for confounding factors, including obesity, menopausal status, and reproductive history, could contribute to heterogenous findings. Further, differences in the methodology of meta-analyses, including different criteria for study inclusion and different statistical approaches, could contribute to inconsistent results.

While current meta-analyses are limited by their inability to measure DDT/DDE exposure at key windows of mammary gland development, two prospective studies examined the impact of early DDT exposure on breast cancer risk. These studies report that elevated concentrations of DDT—but not DDE—are associated with a five-fold increase in breast cancer risk, particularly among women exposed to DDT before puberty (22, 23). Unfortunately, these prospective studies did not investigate the clinical or pathological characteristics of the tumors that are associated with obesogen exposure.

Together, these findings suggest that BPA and/or DDT exposure during critical developmental stages increase breast cancer risk. While early-life exposure to obesogens is known to disrupt mammary gland development, exposure during adulthood may also contribute to breast cancer risk. In premenopausal women, the breast undergoes morphological changes each month under the influence of fluctuating concentrations of estrogen and progesterone during the menstrual cycle. Cumulative exposure to ovarian hormones during the menstrual cycle is a well-established risk factor for breast cancer development (75, 76). Chronic exposure to BPA and DDT/DDE in adulthood, through contaminated food, water, and consumer products, may mimic these hormone-driven pathways, potentially affecting adipocyte-epithelial interactions during breast remodeling to influence breast cancer risk. Another critical window of breast development that may be influenced by obesogens is the pregnancy/lactation/involution period, during which the breast undergoes extensive remodeling under hormonal regulation. This period is marked by dramatic changes in the adipocyte compartment, including the depletion of adipocytes during lactation and their replenishment during mammary gland involution. Disruptions in breast remodeling in premenopausal women might explain the increased risk of TNBC observed in young women with obesity (7–9). However, the specific effects of obesogens on adipocytes during these critical post-pubertal stages of breast development, and how changes in adipocyte function may contribute to breast cancer risk, remains underexplored.

In women with breast cancer, BPA and DDT may also drive the development of more aggressive disease. Ovarian hormones—estrogen and progesterone—are known to promote more aggressive, proliferative tumor phenotypes (77, 78). As such, the xenoestrogenic properties of BPA and DDT (79) may drive tumor aggressiveness in HR-positive subtypes. The prevalence of BPA and DDT in the environment, their ability to cross the placenta (36, 41), and their presence in mother’s milk (37, 42, 43), makes it crucial to understand the long-term risks associated with exposure to elucidate mechanisms that contribute to breast cancer risk and the development of aggressive disease. Future large-scale, longitudinal studies that account for key factors such as menopausal status, reproductive history, and obesity, and interpret results in light of BPA and DDT/DDE concentrations during critical windows of breast development, are required. These studies should also collect information on tumor subtypes to better understand the relationship between obesogen exposure and cancer aggressiveness.

## 4 Obesogens promote adipogenesis and pro-inflammatory phenotypes in adipocytes

Alterations in adipogenesis, lipogenesis, and adipokine secretion are intricately linked to the development of obesity, and may represent mechanisms through which obesogens increase breast cancer risk. Adipogenesis is the process by which mesenchymal stem cells differentiate into mature adipocytes, regulated by transcription factors peroxisome proliferator-activated receptor-γ (PPAR-γ) and C/EBPα (80). This process plays a role in obesity by increasing the number and size of adipocytes, leading to excess fat accumulation and altered metabolic profiles (81, 82). Critically, emerging research implicates the adipogenesis pathway in cancer progression (83). In the tumor microenvironment, tumor-derived exosomes and WNT signaling stimulate de-differentiation of adipocytes into cancer-associated adipocytes, through modulation of PPAR-γ and C/EBPα function (84). Cancer-associated adipocytes promote breast cancer progression through secretion of adipokines and inflammatory cytokines, IL-6, IL-1β, and TNFα (85–87); and release of fatty acids, which are captured by breast cancer cells to support their increased metabolic demands (88, 89).

While the tumor microenvironment can activate adipocytes to drive breast cancer progression, obesogens have also been implicated in modulating the adipogenesis pathway. BPA treatment of preadipocytes increases PPAR-γ and C/EBPα expression (90), promotes differentiation into mature adipocytes (91–93), and increases lipid accumulation (90, 92–94) *in vitro*. In rodents, *in utero* exposure to BPA impairs mammary fat-pad development, through increasing expression of PPAR-γ and accelerating fat-pad maturation (95). Critically, BPA-exposed adipocytes show increased expression of inflammatory cytokines, IL-6, IL-1β, and TNFα (96), which are known to activate key oncogenic pathways in breast cancer cells, such as STAT3 and NF-κB (97). These pathways promote epithelial cell proliferation and survival, driving tumor development. Although direct evidence linking adipocyte-derived cytokines to tumor progression in the context of BPA exposure is limited, these findings could suggest a potential mechanism through which BPA-altered adipocytes contribute to oncogenic transformation and tumor progression in the breast.

Exposure to DDT and/or DDE has a similar effect on adipogenesis. Treatment of pre-adipocytes with DDT and DDE increases expression of PPAR-γ and C/EBPα (98, 99), induces differentiation into mature adipocytes (17, 98, 100) and promotes lipid accumulation (101). While treatment with DDT and/or DDE promotes expression of inflammatory cytokines (IL-6, IL-1β, and TNFα) in other cell types (102–104), the effects of DDT/DDE on adipocytes remains unknown. In humans, plasma concentrations of DDT and DDE positively correlate with inflammatory markers, IL-6, IL-1β, and TNFα (105). These cytokines may promote mammary tumorigenesis through activating STAT3 and NF-κB signaling pathways suggesting a potential link between DDT/DDE exposure and breast cancer development. However, further research is required to identify whether DDT/DDE-exposed adipocytes express inflammatory cytokines and how this impacts mammary tumorigenesis.

Together, these observations are consistent with a role for BPA and DDT/DDE in modulating adipogenesis, which promotes formation of adipocytes with a pro-inflammatory phenotype. The resulting inflammatory milieu might contribute to breast cancer progression through creating an immune microenvironment supportive of tumor growth (85). Specifically, TNBC tumors are enriched for metabolism-related genes (83) and are highly immunogenic (27); phenotypes which might be promoted by the milieu associated with adipogenesis (83, 106). These findings are consistent with the observation that adipogenesis correlates with a worse prognosis in TNBC (83), suggesting potential mechanisms through which obesogens impact TNBC progression. However, while these observations suggest adipogenesis as a potential mechanism through which BPA and DDT/DDE might promote the development of TNBC, direct evidence of causality is lacking. Further research is required to determine whether obesogen-driven changes in adipogenesis influence TNBC development.

## 5 Obesogens promote adipocyte lipogenesis and fatty acid secretion

Another mechanism that may support development of aggressive tumor subtypes is *de novo* fatty acid synthesis; a process which provides essential lipids for rapidly proliferating cells (107, 108). *De novo* fatty acid synthesis is regulated by enzymes, ATP citrate lyase (ACLY), Acetyl-CoA Carboxylase (ACC), and Fatty Acid Synthase (FASN), under the control of transcription factor SREBP-1c (109). Together, these enzymes catalyze the conversion of citrate into fatty acids, which are used for production of triglycerides (109, 110).

Emerging research implicates a role for fatty acid synthesis in cancer (109–112). In breast cancer, increased activity of *de novo* lipid synthesis is an early event for progression from *in situ* to invasive disease (113, 114). Further, expression of lipogenic enzymes, ACLY (115), ACC and FASN (113), are increased in invasive breast cancer, compared to normal breast tissue, where their expression positively correlates with worse clinical outcomes (115–117).

Obesogens are suggested to activate *de novo* lipogenesis in adipose tissue. Exposure of mice to BPA in drinking water increases adipocyte expression of lipogenic enzymes ACC (48) and FASN (48, 90) and SREBP-1c (48, 90), suggesting increased *de novo* synthesis. In humans, BPA exposure is associated with increased serum triglyceride (46, 118, 119), consistent with metabolic alterations and release of triglycerides from adipocytes. Similarly, DDT/DDE also enhances lipogenesis in adipocytes. Treatment of preadipocytes with DDT or DDE increases expression of ACC (99), FASN (99, 101) and SREBP-1c (101). Further, serum concentrations of DDT/DDE positively associate with serum triglycerides in humans (47, 120), consistent with increased *de novo* lipogenesis.

Together, these studies suggest that exposure to BPA and DDT accelerates adipocyte fatty acid synthesis. An enhanced capability of adipocytes to synthesize and secrete fatty acids may promote tumor growth, not only by supplying tumor cells with necessary metabolic resources needed for rapid proliferation, but also by initiating signaling pathways in mammary epithelial cells to drive proliferation (121). Critically, TNBC overexpresses genes involved in metabolism of exogenous-derived fatty acids (122, 123), suggesting that increased fatty acid synthesis in mammary adipocytes support the growth of TNBC. However, studies linking obesogen-induced fatty acid synthesis to TNBC development are lacking. Further research is required to address whether these metabolic changes directly contribute to TNBC growth and progression.

## 6 Obesogens alter adipocyte adipokine expression to promote inflammation

Adipocytes secrete adipokines, which are hormones or cytokines that regulate metabolic function. Among these are adiponectin, which enhances insulin sensitivity and exhibits anti-inflammatory properties; and leptin, often termed the ‘satiety hormone,’ which regulates energy balance by inhibiting hunger, and possesses inflammatory properties (124). In obesity, concentrations of adiponectin decrease, contributing to insulin resistance and metabolic dysfunction (125, 126). Conversely, concentrations of leptin are elevated during obesity, leading to leptin resistance, reducing its effectiveness in appetite suppression (127, 128). Altered adiponectin and leptin concentrations are associated with increased inflammation in obesity (129).

Adipokines may play a role in cancer development. Decreased serum adiponectin correlates with increased inflammation, and an increased risk of breast cancer development (130–132). In breast cancer patients, low serum adiponectin associates with more aggressive tumors, including larger size and higher grade (132). Similarly, leptin promotes inflammation (124) and positively correlates with increased breast cancer risk (133–135). In breast cancer patients, serum leptin associates with worse clinical features (133), and is highest in TNBC, compared to HR-positive tumors (134). Together, these findings suggest that alterations in adipokine secretion might favor the development of more aggressive, triple-negative, tumors.

Obesogens are suggested to influence adipokine expression. *Ex vivo*, BPA treatment of breast adipose explants suppresses adiponectin release (136). In rodents, perinatal BPA exposure reduces adiponectin concentrations in serum and adipose tissue (137). Further, in humans, serum BPA negatively correlates with adiponectin concentrations (138–140), and positively correlates with leptin (138, 140).

While the effect of DDT/DDE on adipokine expression is less studied, one study reported an association between prenatal exposure to DDE with decreased serum adiponectin (141). Despite the lack of studies of DDT/DDE on adipokine expression, the established influence of these chemicals on obesity indirectly implies a potential effect on adipokine expression in the mammary gland. Research investigating the effect of DDT/DDE exposure on adiponectin and leptin is warranted, to clarify their roles in metabolic dysregulation and associated health risks.

Together, these results suggest that BPA and DDT reduce adiponectin and increase leptin expression in adipose tissue; alterations that are independently linked to the development of more aggressive, triple-negative tumors. Consequently, obesogens may promote TNBC development, through disruption of the adipokine milieu in the mammary gland. In addition to altering adipokine profiles, BPA can also directly affect the surrounding immune microenvironment, through altering T cells, B cells, macrophages and dendritic cells (50, 142), to promote a pro-inflammatory microenvironment that may be particularly supportive of TNBC development. Currently, studies linking obesogen-induced changes in adipokine secretion profiles to TNBC development are lacking. Further research is required to address whether these metabolic changes directly contribute to TNBC growth and progression, and to identify underlying mechanisms that could inform therapeutic strategies.

## 7 Conclusion

**Figure 1:**
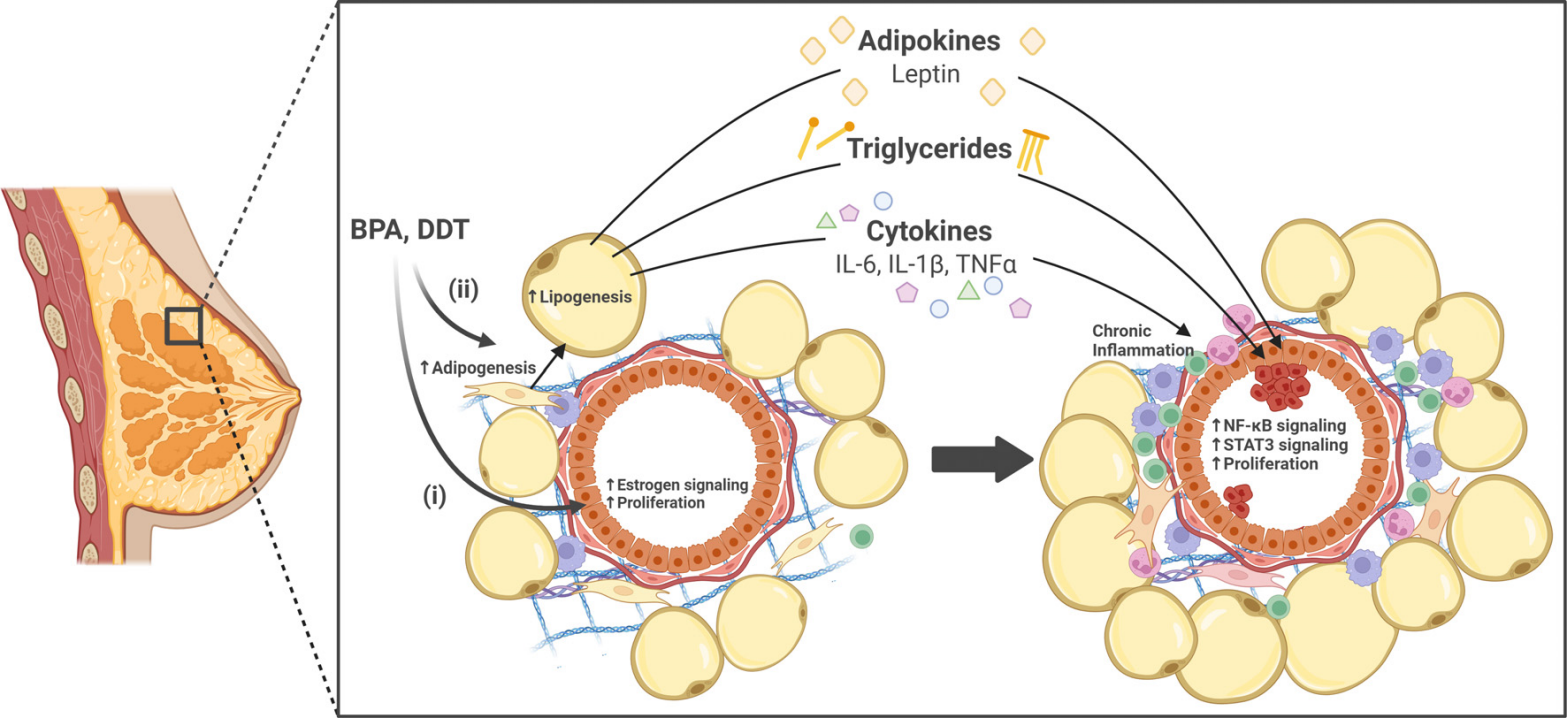
The effects of obesogens on the breast. Obesogens bisphenol A (BPA) and dichlorodiphenyltrichloroethane (DDT) are suggested to contribute to breast cancer development through two pathways; (i) directly through their estrogenic effects on mammary epithelial cells, or (ii) indirectly through their impact on adipocyte function. Specifically in adipocytes, BPA and DDT promote adipogenesis, increase lipogenesis, and increase secretion of adipokines (e.g., leptin), cytokines (e.g., IL-6, IL-1β, TNFα), and triglycerides. These adipokines and cytokines contribute to chronic inflammation, and are known to activate oncogenic pathways in mammary epithelial cells, such as NF-κB and STAT3, leading to epithelial cell proliferation. Together, these processes may contribute to oncogenic transformation and breast cancer progression. Created in BioRender.com.

The prevalence of obesogens in our environment remains a significant concern. While research has begun to elucidate the estrogen-mediated mechanisms through which these chemicals disrupt mammary gland development, the role of obesogens in altering the function of mammary adipocytes and their contribution to epithelial cell growth and the development of aggressive breast cancer subtypes remains unclear. Obesogens affect adipogenesis, and lipogenesis, and alter the adipokine secretion profile, contributing to breast cancer risk and progression by creating a pro-inflammatory, nutrient-rich environment that can promote oncogenic transformation. Critically, the cumulative effects of lifelong exposure to multiple obesogens may amplify these disruptions, further increasing breast cancer risk. The effects of obesogens on the breast are summarized in **Figure 1**. Understanding how obesogens drive breast cancer development is crucial for informing updated health guidelines to reduce exposure and mitigate associated risks. Such actions would reduce the impact of environmental chemicals on global breast cancer incidence, contributing to improved outcomes for women.
